# Leptin promotes migration and invasion of breast cancer cells by stimulating IL-8 production in M2 macrophages

**DOI:** 10.18632/oncotarget.11761

**Published:** 2016-08-31

**Authors:** Hong Cao, Yunxiu Huang, Lin Wang, Hong Wang, Xueli Pang, Kuangfa Li, Weiqi Dang, Hao Tang, Lan Wei, Min Su, Cuiping Tang, Tingmei Chen

**Affiliations:** ^1^ Department of Laboratory Medicine Key Laboratory of Diagnostic Medicine, Ministry of Education Chongqing Medical University, Chongqing, China

**Keywords:** breast cancer, IL-8, invasion, leptin, migration

## Abstract

This study aims to investigate the mechanisms underlying leptin-mediated crosstalk between tumor-associated macrophages (M2 macrophages) and breast cancer cells. THP1 human leukemic monocytes were induced to differentiate into M2 macrophages by PMA (100 nM) and IL-4 (20 ng/mL). Quantitative RT-PCR and Western blot revealed that leptin (100 nM) significantly increased the expression of leptin receptor (ObR) in the M2 macrophages (*P* < 0.01) and stimulated interleukin (IL)-8 expression in the M2 macrophages, mouse macrophage cells RAW264.7, and primary mouse peritoneal macrophages in a dose- and time-dependent manner. Leptin-induced IL-8 production was sensitive to the ERK inhibitor PD980590 (10 μmol/L), p38 MAPK inhibitor SB203580 (20 μmol/L), and anti-ObR neutralizing antibody (4 μg/mL). Leptin (100 ng/mL) substantially increased the phosphorylation of p38 and ERK1/2. Thus, leptin may induce IL-8 production in M2 macrophages by interacting with ObR to activate the p38 and ERK signaling pathways. Scratch and transwell chamber assay showed that both recombinant IL-8 and leptin-induced M2 macrophage-derived IL-8 promoted the migration and invasion of human breast cancer cells MCF7 and MDA-MB-231 (All *P* < 0.01). In a nude mice xenograft model of breast cancer (*n* = 5 per group), injection of leptin (0.1 μg/g) dramatically increased tumor volume and mass, reduced survival, exacerbated pulmonary metastasis, and elevated IL-8 and Ki67 expression in the tumor tissue (All *P* < 0.05) compared with PBS injection. Depletion of mouse macrophage by Clophosome^®^-clodronate liposome and injection of anti-mouse IL-8 neutralizing antibodies in the xenograft tumor significantly attenuated those leptin-mediated stimulations (All *P* < 0.05). These findings indicate that leptin may promote tumor growth and metastasis by stimulating IL-8 production in tumor-associated macrophage.

## INTRODUCTION

Obesity and high adipose tissue mass, which increase the risk of aggressive tumor phenotype and worsen survival of breast cancer [1–5], can cause excessive secretion of leptin and other pro-inflammatory factors [6–8]. High leptin levels have been found to increase breast cancer risk [9–14]. Leptin levels and the expression of leptin receptor (ObR) are substantially higher in breast cancer cells and tissues than in normal mammary epithelial cells and tissues [9–11]. Women with increased levels of serum leptin often have a higher incidence of breast cancer than women with normal leptin levels [12–14]. Leptin regulates cell behaviors by binding to ObR to induce canonical (JAK2/STATs, PI-3K/AKT, MAPK/ERK 1/2) and non-canonical signaling pathways (p38, JNK, PKC, MAPK and AMPK) [15–17]. High levels of serum leptin may lead to aberrant intracellular signaling, which consequently results in cancer development [15, 18].

The role of leptin in mediating the crosstalk between the tumor microenvironment and breast cancer cells remains unclear. Tumor-associated macrophages (M2 macrophages), which are the most notable hematopoietic cells in the tumor microenvironment, have been found to promote breast cancer progression and correlate with poor prognosis [19–25]. M2 macrophages can secret cancer-promoting cytokines, growth factors, and inflammatory factors [26, 27]. Leptin receptor ObRb has been found to be expressed in macrophages [28]. Leptin induces macrophage recruitment to the tumor microenvironment by stimulating aromatase and estrogen receptor (ER) α in the macrophages [29]. In addition, leptin also stimulates macrophages to secret vascular endothelial growth factor and pro-inflammatory cytokines, including interleukin (IL)-1, TNF-α, and IL-6 [15, 19]. Thus, leptin may induce the release of cancer-promoting factors from M2 macrophages to indirectly stimulate breast cancer progression. This study aims to test this hypothesis and further investigate the molecular mechanism underlying leptin-mediated cancer promoting effects of M2 macrophages on breast cancer cells.

## RESULTS

### Leptin induced ObR expression and IL-8 production in M2 macrophages

**Figure 1:**
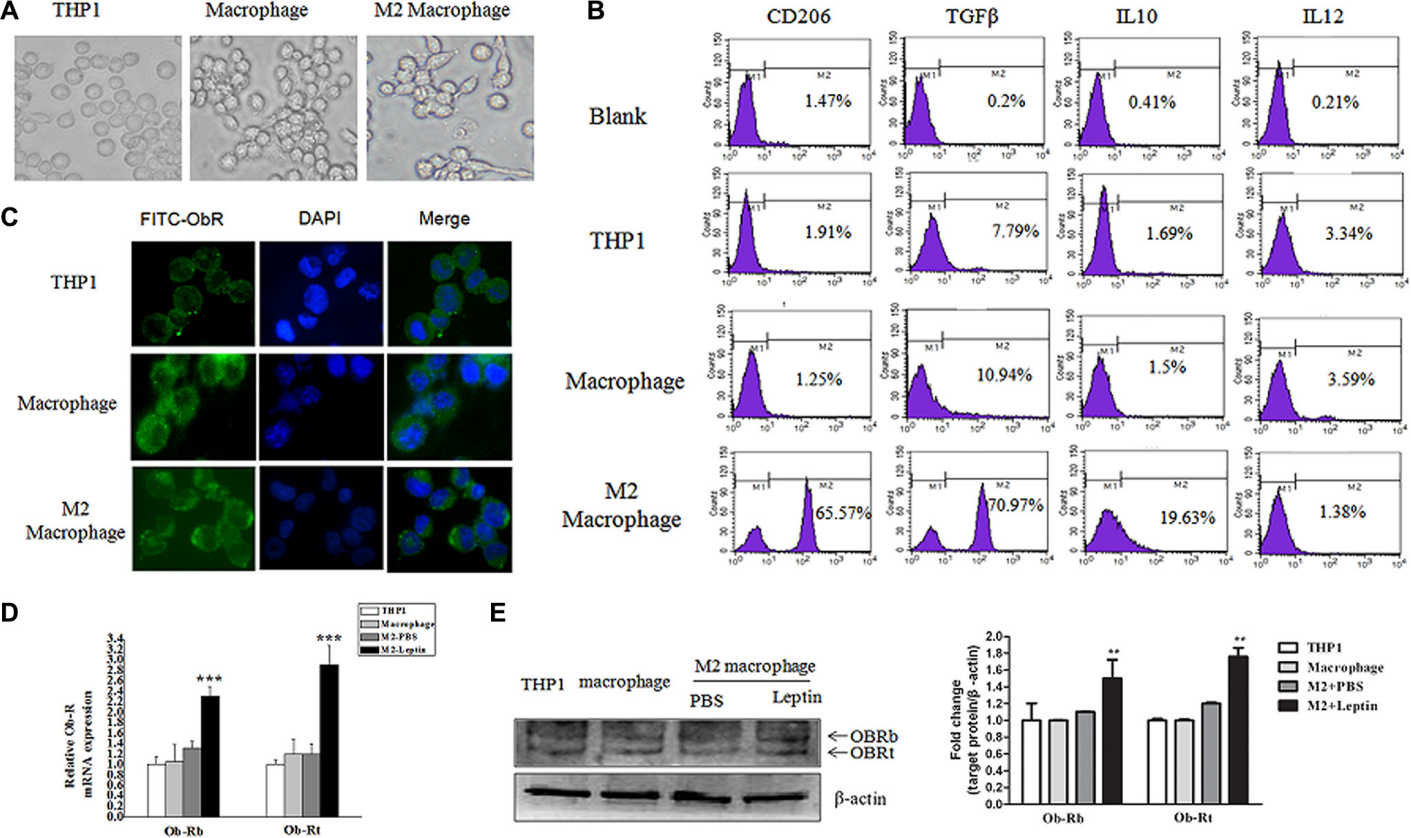
Leptin stimulated ObR expression in M2 macrophages THP1 cells were treated with PMA (100 nM, 72 hours) plus IL-4 (20 ng/mL, 36 hours) to induce M2 macrophage differentiation. (**A**) Representative phase contrast images of THP1 cells, THP1 macrophages, and M2 macrophages. (**B**) Flow cytometry analysis of the expression of CD206, TGF-β, IL-10, and IL-12 in THP1 cells, THP1 macrophages, and M2 macrophages. (**C**) Representative images of immunofluorescence staining for ObR in THP1, THP1 macrophages, and M2 macrophages. Images are at magnification of 400×. (**D**) qRT-PCR analysis of the mRNA level of long form (ObRb) and short form (ObRt) leptin receptor in THP1, THP1 macrophage, and M2 macrophages treated with PBS or leptin. (**E**) A representative image of Western blot and densitometry analysis showing the expressions of ObRb and ObRt in THP1, THP1 macrophages, and M2 macrophages. **represent significant difference between the M2 + leptin group versus the M2 + PBS group, *P* < 0.01.

The morphology of THP1 cells was changed from round into anchorage-dependent shape after the cells differentiated into macrophages (Figure 1A). Flow cytometry revealed that M2 macrophages, which were induced from THP1 cells by PMA (100 nM) and IL-4 (20 ng/mL), expressed lower levels of IL-12 (1.38%) and higher levels of TGF-β (70.97%) and IL-10 (19.63%) compared with THP1 cells and THP1 macrophages (Figure 1B). The expression of the mannose receptor CD206, a M2 macrophage surface marker, was dramatically increased in M2 macrophages (Figure 1B), suggesting that exposure to PMA and IL-4 appears to successfully induce THP1 cells to differentiate into M2 macrophages. Immunofluorescence staining showed that ObR was expressed weakly in THP1 cells, whereas highly expressed in both THP1 macrophages and M2 macrophages (Figure 1C). Leptin (100 nM) significantly increased mRNA (Figure 1D) and protein expression (Figure 1E) of both the long form (ObRb) and the short form (ObRt) leptin receptor in M2 macrophages (All *P* < 0.01).

**Figure 2:**
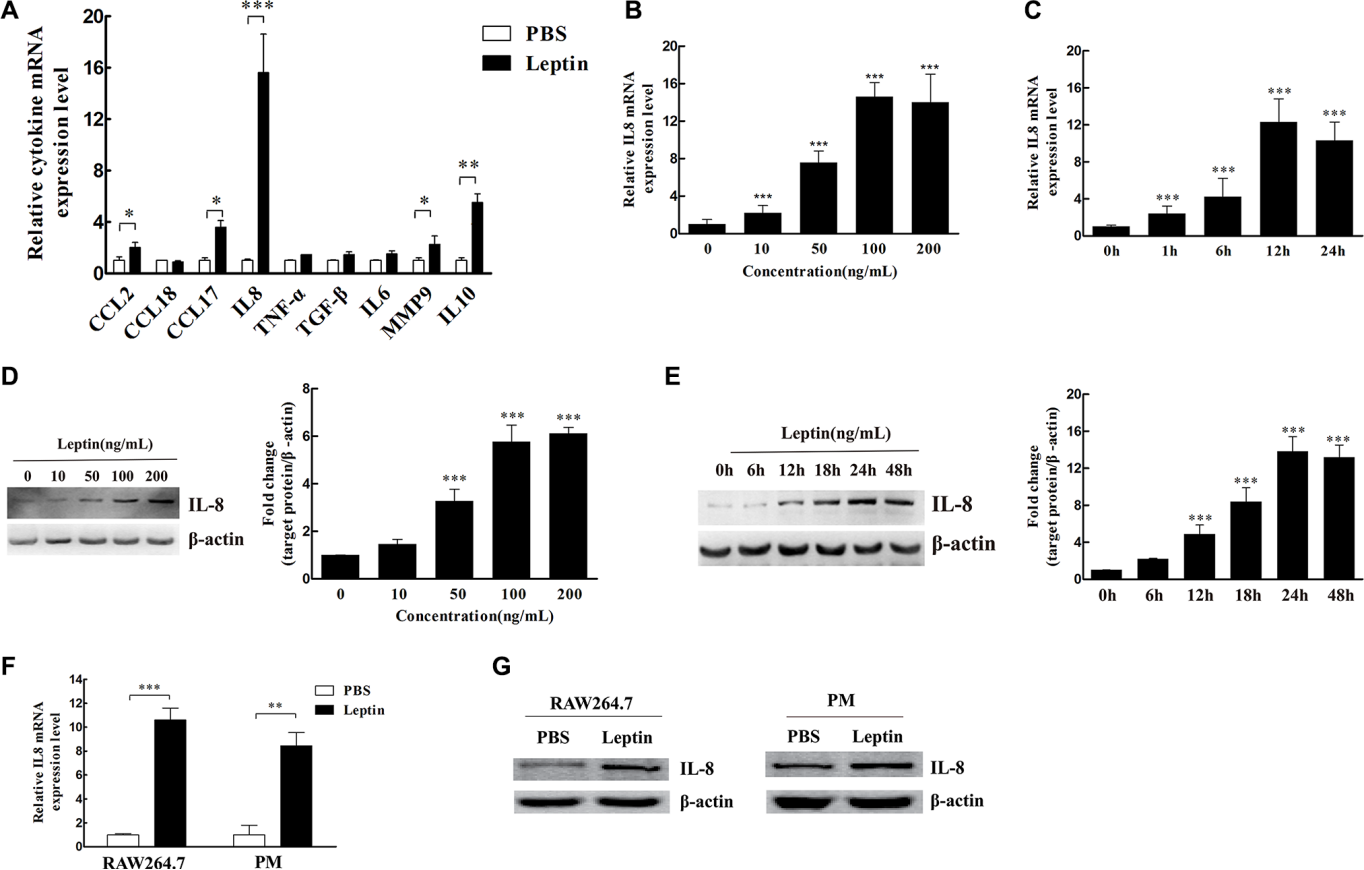
Leptin induced IL-8 production in M2 macrophages (**A**) The relative mRNA levels of cytokines in M2 macrophages treated with leptin or PBS. *P < 0.05; **P < 0.01; ***P < 0.001. (**B**) The dose effect of leptin (0–200 ng/mL) on IL-8 mRNA expression in M2 macrophages. ***represents significant difference between the indicated groups versus the 0 ng/mL group, *P* < 0.001. (**C**) The time course (0 to 24 hours) of IL-8 mRNA expression in M2 macrophages treated with 100 ng/mL leptin. ***represents significant difference between the indicated groups versus the 0 h group, *P* < 0.001. (**D**) The dose effect of leptin (0–200 ng/mL) on IL-8 protein expression in M2 macrophages. A representative Western blot image and densitometry analysis are presented. β-actin was used as the loading control. ***represents significant difference between the indicated groups versus the 0 ng/mL group, *P* < 0.001. (**E**) The time course (0 to 48 hours) of IL-8 protein expression in M2 macrophages treated with 100 ng/mL leptin. β-actin was used as the loading control. A representative Western blot image and densitometry analysis are presented. ***represents significant difference between the indicated groups versus the 0 h group, *P* < 0.001. (**F**) IL-8 mRNA expression in mouse macrophage cells RAW264.7 and mouse peritoneal macrophages (PM) treated with 100 ng/mL leptin for 12 h. **P < 0.01, ***P < 0.001. (**G**) IL-8 protein expression in RAW264.7 cells and mouse peritoneal macrophages treated with 100 ng/mL leptin for 48 h. A representative Western blot image is presented.

Leptin (100 ng/mL) increased IL-8 mRNA expression (16-fold) the most in M2 macrophages compared with other cytokines (Figure 2A). Leptin induced IL-8 mRNA expression in a dose- (Figure 2B) and time- (Figure 2C) dependent manner (All *P* < 0.001). Leptin-stimulated IL-8 protein expression was also in a dose- (Figure 2D) and time- (Figure 2E) dependent manner (All *P* < 0.001). The optimal dose of leptin and time for maximal IL-8 induction was 100 ng/mL and 24 hours of treatment, respectively. In addition, leptin (100 ng/mL) also significantly increased IL-8 mRNA (*P* < 0.01, Figure 2F) and protein expression (Figure 2G) in RAW246.7 cells and primary mouse peritoneal macrophages (PM).

### Both recombinant IL-8 and IL-8 from Leptin-treated M2 macrophages increased the migration and invasion of breast cancer cells

**Figure 3:**
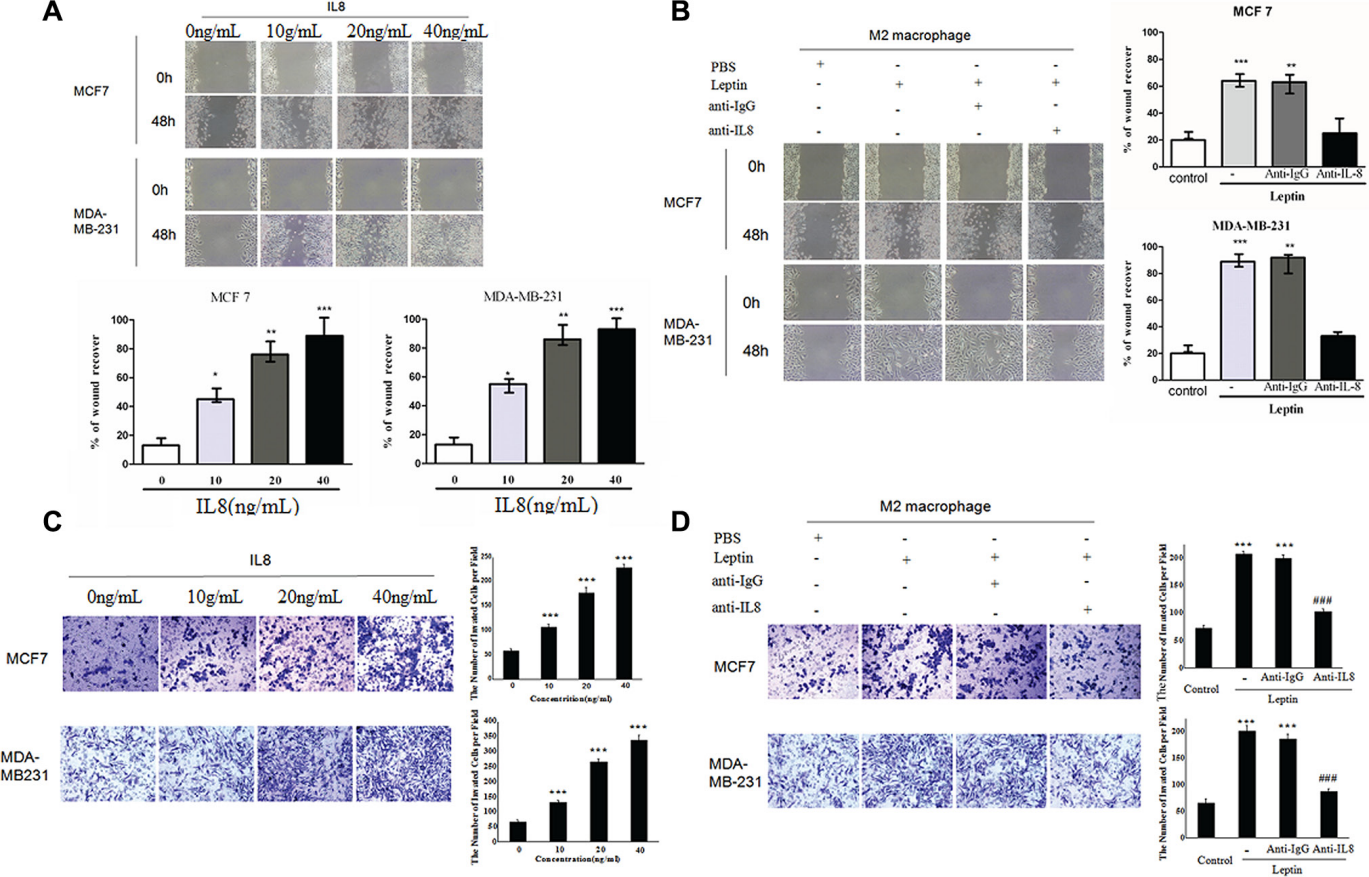
Recombinant IL-8 and IL-8 from leptin-treated M2 macrophages stimulated the migration and invasion of breast cancer cells MCF7 and MDA-MB-231 (**A**) Scratch assay on MCF7 and MDA-MB-231 cells treated with recombinant IL-8 (0–40 ng/mL). The cells were treated for 48 hours. Phase contrast images are at a magnification of 100×. *represents significant difference between the indicated groups versus the 0 ng/mL group. *P < 0.05, ** *P* < 0.01, ***P < 0.001. (**B**) Scratch assay on MCF7 and MDA-MB-231 cells in the co-culture with M2 macrophages. The breast cancer cells were cultured in the lower chambers and leptin-treated or PBS-treated M2 macrophages were cultured in the upper chambers. The co-culture was incubated for 48 hours. Anti-IL-8 antibody (1 μg/mL) or an isotype-matched IgG control (IgG) at the same concentration was added in the upper chambers. ***represents significant difference between the Leptin group and the control group, *P* < 0.001. **represents significant difference between the Leptin + anti-IgG group and the Leptin + anti-IL-8 group, *P* < 0.01. (**C**) Transwell chamber invasion assay on MCF7 and MDA-MB-231 cells treated with recombinant IL-8 (0–40 ng/mL). Phase contrast images are at a magnification of 200×. ***represents significant difference between the indicated groups versus the 0 ng/mL group, *P* < 0.001. (**D**) Transwell chamber invasion assay on MCF7 and MDA-MB-231 cells in the co-culture with M2 macrophages. The breast cancer cells were cultured in the upper chambers and leptin-treated or PBS-treated M2 macrophages were cultured in the lower chambers. The co-culture was incubated for 48 hours. Anti-human IL-8 antibody (1 μg/mL) or an isotype-matched IgG control (IgG 1 μg/mL) was added in the lower chambers. Phase contrast images are at a magnification of 200×. ***represent significant difference between the indicated groups versus the control group, *P* < 0.001. ###represent significant difference between the Leptin + anti-IgG group and the Leptin + anti-IL-8 group, *P* < 0.001.

Recombinant IL-8 significantly increased the migration (*P* < 0.05, Figure 3A) and invasion (*P* < 0.001, Figure 3C) of MCF7 and MDA-MB-231 cells in a dose-dependent manner (0–40 ng/mL). Co-culture with leptin-treated M2 macrophages significantly stimulated the migration (*P* < 0.01, Figure 3B) and invasion (*P* < 0.001, Figure 3D) of MCF7 and MDA-MB-231 cells. Functional neutralizing antibody against IL-8 significantly blocked the co-culture-induced migration (*P* < 0.01, Figure 3B) and invasion (*P* < 0.01, Figure 3D) of MCF7 and MDA-MB-231 cells. These data suggest that IL-8 from leptin-treated M2 macrophages may stimulate breast cancer cell migration and invasion.

### Leptin induced IL-8 production by interacting with ObR to activate MAPK/ERK 1/2 and P38/MAPK signaling pathways in M2 macrophages

**Figure 4:**
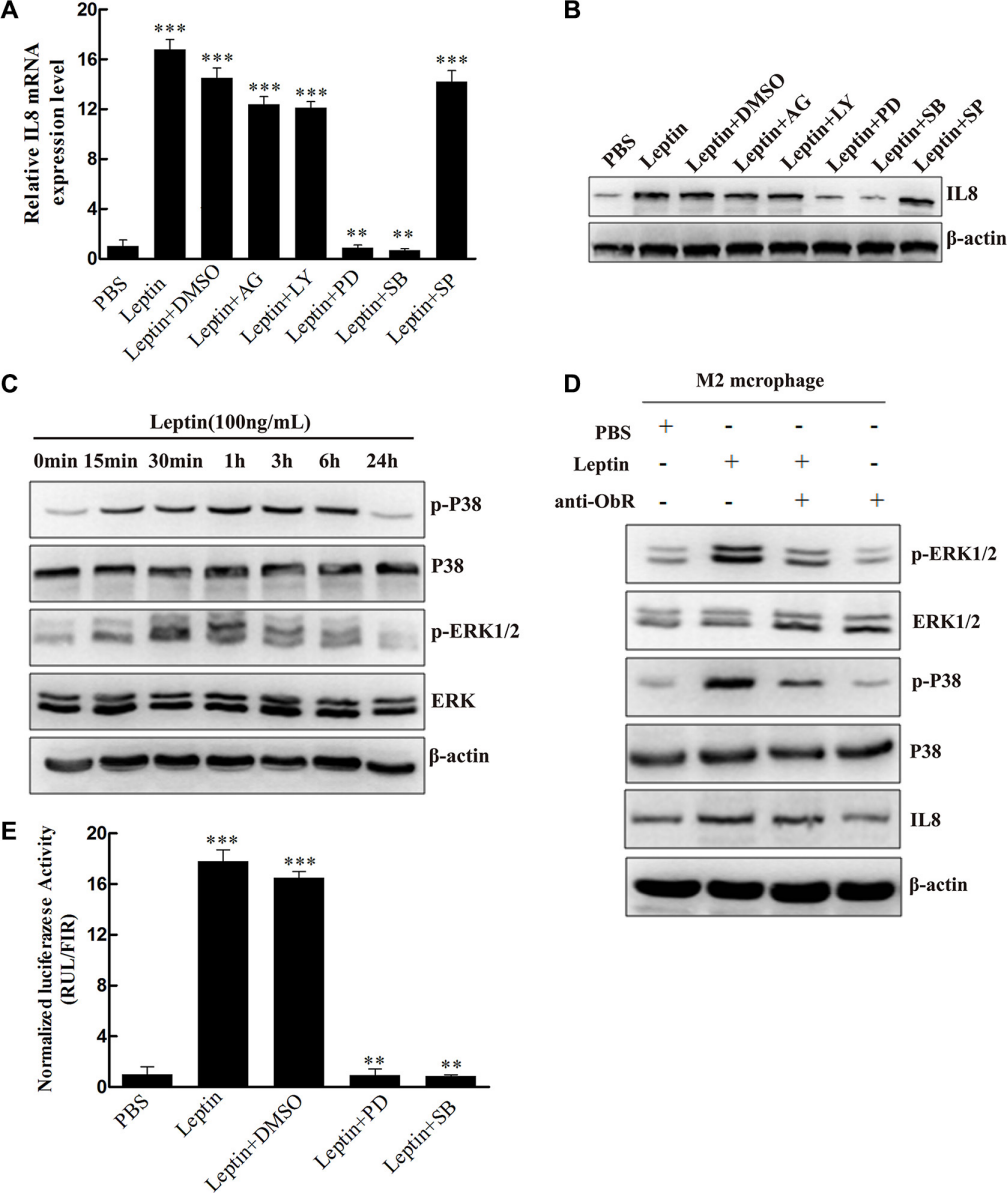
Leptin induced IL-8 production in M2 macrophages by activating the MAPK/ERK 1/2 and P38/MAPK signaling pathways in an ObR-dependent manner (**A**) Relative IL-8 mRNA expression in M2 macrophages treated with leptin with or without the inhibitors. M2 macrophages were pretreated with JAK inhibitor AG490 (50 μmol/L), PI3K inhibitor LY294002 (10 μmol/L), ERK inhibitor PD980590 (10 μmol/L), p38 MAPK inhibitor SB203580 (20 μmol/L), or JNK inhibitor SP600125 (50 μmol/L) for 1 hour and then were treated with PBS or leptin (100 ng/mL). Relative IL-8 mRNA levels were analyzed by qRT-PCR. ***represents significant difference between the indicated groups versus the PBS group, *P* < 0.001. **represents significant difference between the indicated groups versus the Leptin + DMSO group, *P* < 0.01. (**B**) A representative image of Western blot for IL-8 protein expression in M2 macrophages treated in the same manner as (A). (**C**) A representative image of Western blot showing the time course of leptin-induced p38 and ERK 1/2 phosphorylation. M2 macrophages were treated with leptin (100 ng/mL) for 0–24 hours. (**D**) A representative image of Western blot showing leptin-induced ObR-dependent phosphorylation of ERK 1/2 and p38 and production of IL-8. M2 macrophages were pretreated with a polyclonal anti-human ObR antibody (4 μg/mL) for 16 hours and then treated with PBS or leptin (100 ng/mL). (**E**) Luciferase reporter assay to measure leptin-mediated IL-8 promoter activation. M2 macrophages were pretreated with ERK inhibitor PD980590 (10 μmol/L), p38 MAPK inhibitor SB203580 (20 μmol/L), or JNK inhibitor SP600125 (50 μmol/L) for 1 hour and then were treated with PBS or leptin (100 ng/mL). Relative luciferase units (RLU) normalized to β-galactosidase activity are shown. ***represents significant difference between the indicated groups versus the PBS group, *P* < 0.001. **represents significant difference between the indicated groups versus the Leptin + DMSO group, *P* < 0.01.

To investigate the molecular mechanism underlying leptin-induced IL-8 production in M2 macrophages, we tested the effects of inhibitors of signaling molecules on the IL-8 production. The inhibitors of JAK (AG490: 50 μmol/L), PI3K (LY294002: 10 μmol/L), and JNK (SP600125: 50 μmol/L) had no significant effect on IL-8 mRNA (Figure 4A) and protein expression (Figure 4B) in M2 macrophages. In contrast, both the ERK inhibitor PD980590 (10 μmol/L) and the p38 MAPK inhibitor SB203580 (20 μmol/L) completely blocked Leptin-induced IL-8 mRNA (*P* < 0.01, Figure 4A) and protein expression (Figure 4B), suggesting that ERK and p38 MAPK signaling pathways may be involved in leptin-induced IL-8 production. Indeed, leptin markedly stimulated the phosphorylation of p38 (p-p38) and ERK1/2 (p-ERK1/2) in M2 macrophages, and the stimulation peaked at one hour of leptin treatment (Figure 4C). The total proteins of p38 and ERK1/2 were not affected (Figure 4C). To examine whether leptin-induced phosphorylation of p38 and ERK1/2 could require ObR, we inhibited ObR with a functional neutralizing antibody against ObR. Anti-ObR considerably blocked leptin-induced p38 and ERK1/2 phosphorylation (Figure 4D). Luciferase reporter assay revealed that leptin dramatically activated IL-8 promoter (*P* < 0.001); the ERK inhibitor PD980590 (10 μmol/L) and the p38 MAPK inhibitor SB203580 (20 μmol/L) significantly blocked leptin-induced activation of IL-8 promoter (*P* < 0.01, Figure 4E). These data suggest that leptin may interact with ObR to activate MAPK/ERK 1/2 and P38/MAPK signaling pathways, which in turn activate IL-8 promoter and stimulate IL-8 production in M2 macrophages.

### IL-8, ObR, and CD68 were over-expressed in human invasive breast carcinoma tissue specimens

**Figure 5:**
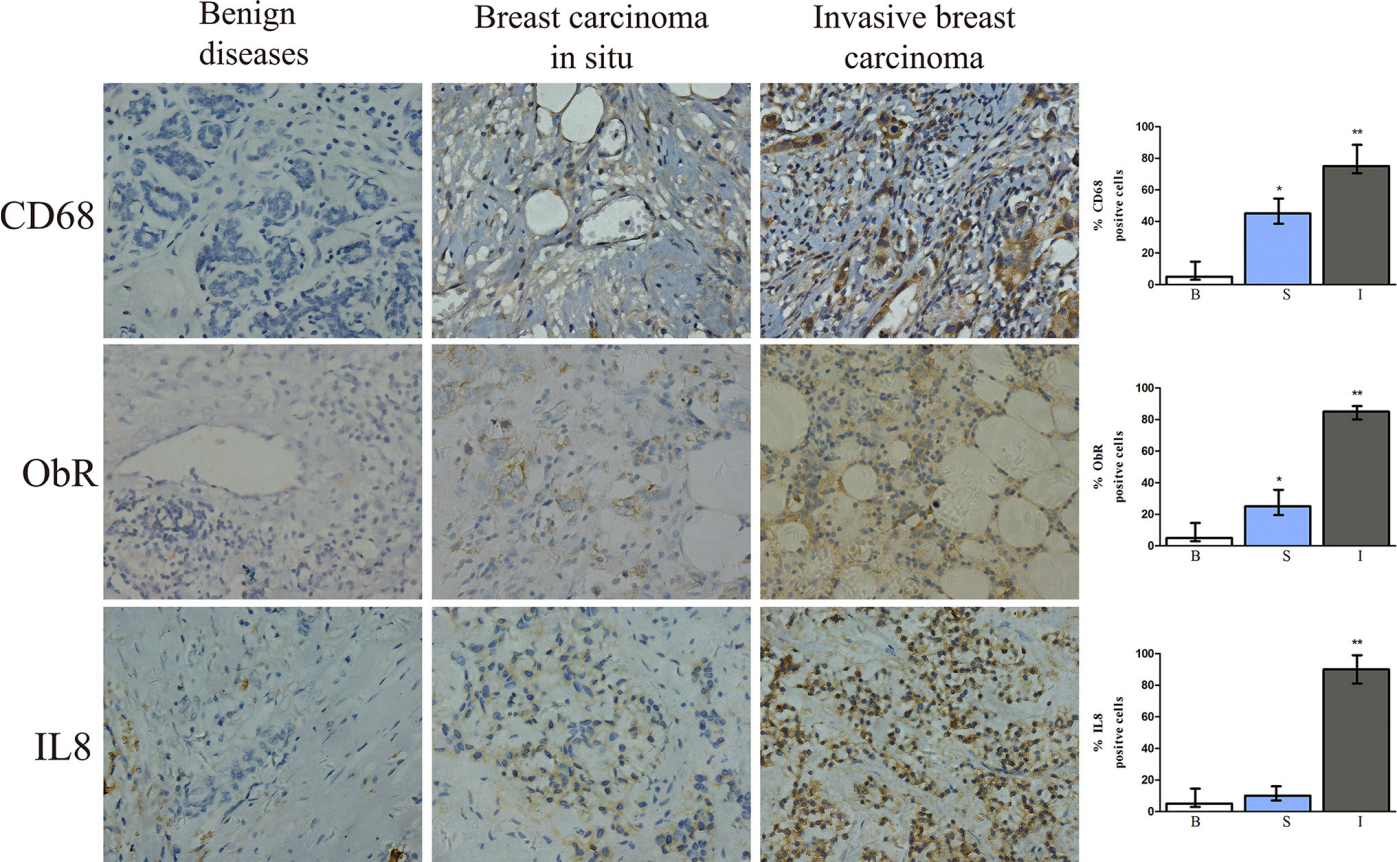
Immunohistochemical staining for CD68, ObR, and IL-8 of human breast tissue specimens Representative images of IHC staining for CD68, ObR, and IL-8 of human breast tissues with benign breast disease, breast carcinoma *in situ*, or invasive breast carcinoma. *represents significant difference between breast carcinoma *in situ* (S) and benign tissue (B), *P* < 0.05. **represents significant difference between invasive breast carcinoma (I) and benign tissue (B), *P* < 0.01.

Immunohistochemistry revealed that ObR, CD68, and IL-8 were significantly higher in invasive breast carcinoma tissue specimens than in benign and carcinoma *in situ* tissue specimens (*P* < 0.05, Figure 5).

### Leptin promoted breast cancer progression via stimulating IL-8 production in macrophage in nude mouse xenograft model

**Figure 6:**
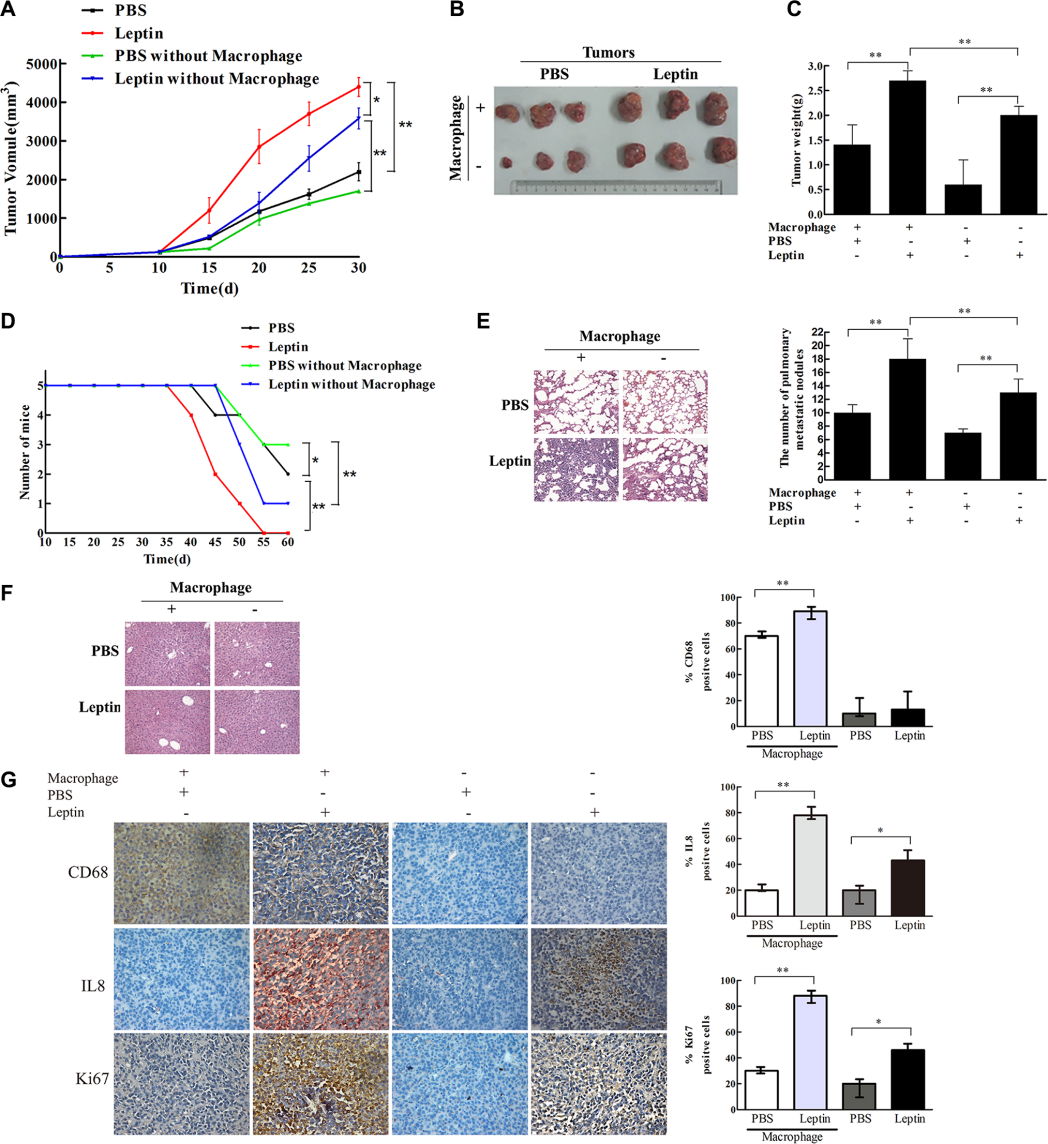
Leptin promoted breast cancer growth and metastasis in mouse xenograft model Breast cancer cells MDA-MB231 (1 × 10^7^ cells) were transplanted to the mammary fat pad of nude mice (5 per group). When the tumor reached a diameter of 0.5 cm, PBS or leptin (0.1 μg/g) was injected into the tumor once every 5 days for 4 weeks. Mice were intraperitoneally injected with Clophosome-Clodronate Liposomes (neutral) or control neutral liposome to deplete macrophages before receiving leptin injection. (**A**) Tumor volume in the PBS, leptin, PBS + macrophage depletion, and leptin + macrophage depletion groups. *P < 0.05, **P < 0.01. (**B**) Photos of tumor xenografts at day 30 after transplantation of human breast cancer cells. (**C**) Tumor wet weight at day 30 after the transplantation. **P < 0.01. **(D**) Mouse survival. *P < 0.05, **P < 0.01. (**E**) Representative images of H & E staining of the lung tissue and the figure showing the number of metastatic nodules in the lung. **P < 0.01. (**F**) Representative images of H & E staining of the liver tissue. (**G**) Representative images of IHC staining of CD68, Ki-67, and IL-8 of the tumor tissue and the figure showing the quantitative analysis. *P < 0.05, **P < 0.01.

**Figure 7:**
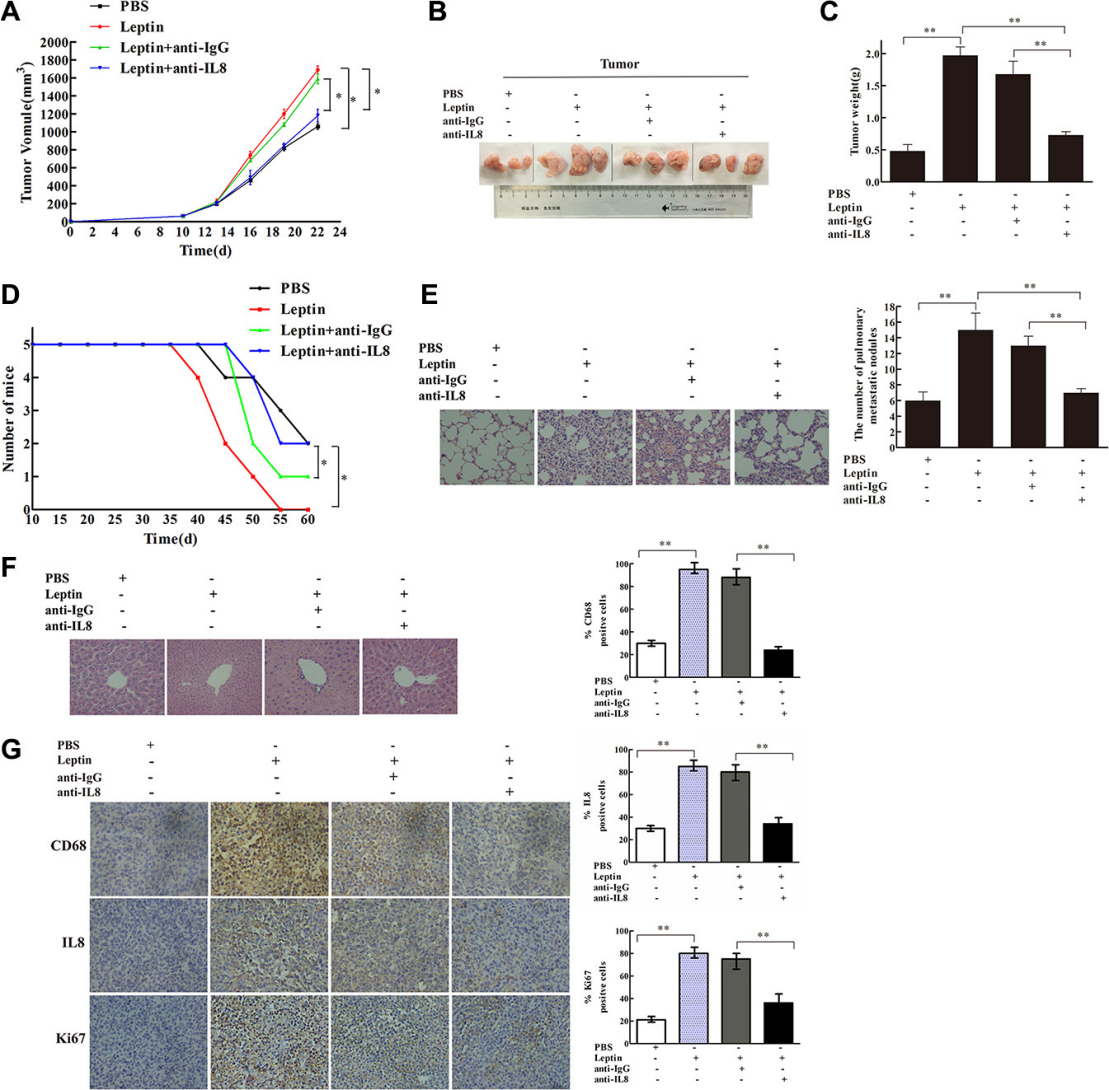
IL-8 neutralization blocked leptin-mediated stimulation of breast cancer growth and metastasis in mouse xenograft model The mouse xenograft model and leptin injection were conducted as in Figure 6. In the leptin-treated group, polyclonal rabbit anti-mouse IL-8 neutralization antibody (Abbexa Ltd, Cambridge, UK) was injected intraperitoneal in the mice at an initial dose of 0.2 mL per mouse and then 0.1 mL per mouse twice per week for 2 weeks. Polyclonal rabbit IgG (Bioss Ltd, Beijing, China) was used as the control. Mice received the injection of leptin and the antibodies for 2 weeks. (**A**) Tumor volume in the PBS, leptin, leptin + anti-IgG, and leptin + anti-IL8 groups. *P < 0.05. (**B**) Photos of tumor xenografts at day 21 after transplantation of human breast cancer cells. (**C**) Tumor wet weight at day 21 after the cell transplantation. **P < 0.01. (**D**) Mouse survival. *P < 0.05. (**E**) Representative images of H & E staining of the lung tissue and the figure showing the number of metastatic nodules in the lung. **P < 0.01. (**F**) Representative images of H & E staining of the liver tissue. (**G**) Representative images of IHC staining of CD68, Ki-67, and IL-8 in the tumor tissue and the figure showing quantitative analysis of the staining. **P < 0.01.

From day 15 to day 30 after leptin injection, mice in the leptin group developed the largest tumor volume (*P* < 0.05, Figure 6A). Compared with PBS injection, leptin injection significantly increased tumor mass on day 30 (*P* < 0.01, Figure 6B and 6C), worsened survival (*P* < 0.05, Figure 6D), exacerbated pulmonary metastasis (*P* < 0.01, Figure 6E), and elevated the expression of IL-8 and Ki67 in the tumor tissue (*P* < 0.01, Figure 6G). In contrast, liver metastasis was not affected by leptin injection (Figure 6F). CD68 expression, a marker for macrophages, was dramatically reduced in the mice treated with Clophosome^®^-clodronate liposome (Figure 6G), indicating an effective depletion of macrophages. Macrophage depletion significantly reduced leptin-induced tumor volume (*P* < 0.05, Figure 6A) and tumor weight (*P* < 0.01, Figure 6C), improved survival (*P* < 0.05, Figure 6D), attenuated leptin-mediated exacerbation pulmonary metastasis (*P* < 0.01, Figure 6E), and decreased leptin-stimulated expression of IL-8 and Ki67 in the tumor (*P* < 0.05, Figure 6G). Similar to the inhibitory effects of macrophage depletion, mice in the anti-mouse IL-8 neutralizing antibodies + leptin group had significantly reduced tumor volume (*P* < 0.05, Figure 7A) and weight (*P* < 0.01, Figure 7B and 7C), improved survival (*P* < 0.05, Figure 7D), attenuated pulmonary metastasis (*P* < 0.01, Figure 7E), and decreased IL-8 and Ki67 expression in the tumor (*P* < 0.01, Figure 7G) compared with mice in the control anti-IgG + leptin group. No liver metastasis was observed in the mice (Figure 7F). These results suggest that leptin may promote breast cancer progression via stimulating IL-8 production in macrophage *in vivo*.

## DISCUSSION

**Figure 8:**
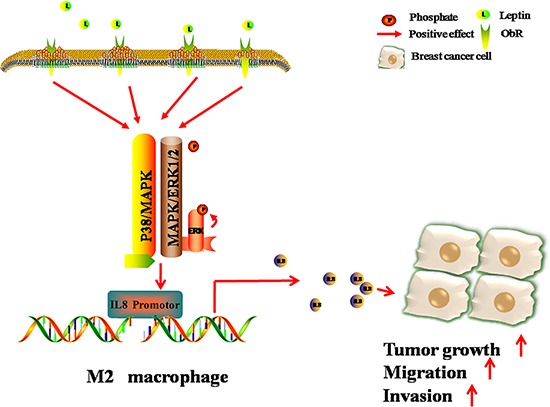
Schematic diagram showing that leptin promotes the migration and invasion of breast cancer cells by stimulating IL-8 production from M2 macrophages The binding of leptin to its receptor ObR results in the phosphorylation of p38 and ERK1/2, consequently activating IL-8 promoter and stimulating IL-8 production in M2 macrophages. IL-8 from M2 macrophages then induces breast cancer progression.

The current study investigated the molecular mechanism underlying leptin-mediated macrophage-breast cancer interaction. The data in the current study suggest that leptin may up-regulate ObR to activate the P38/MAPK and MAPK/ERK 1/2 signaling pathways, which in turn stimulate IL-8 promoter and IL-8 production in M2 macrophages, consequently promoting breast cancer progression (Figure 8).

Binding of leptin to leptin receptors can trigger a series of downstream signaling events, including both canonical (MAPK/ERK 1/2, JAK2/STATs, PI-3K/AKT) and non-canonical signaling pathways (p38 MAPK, JNK, PKC and AMPK), in various types of cells [15]. Our previous study has shown that leptin stimulates IL-8 expression via activating the PI3K/AKT signaling pathway in breast cancer cells MCF7 and SKBR3 [33]. Here, we found that in M2 macrophages, the leptin-ObR interaction activated the p38/MAPK and MAPK/ERK 1/2 signaling pathways but not the PI3K/AKT pathway. In addition, in M2 macrophages, leptin-induced IL-8 expression was mainly at transcriptional level because leptin activated IL-8 promoter rapidly. Our results cannot rule out the possibility that leptin may also increase IL-8 production at translational and/or post-translational levels in M2 macrophages. Thus, leptin-ObR interaction appears to link to distinct intracellular signaling cascades in different types of cells.

In the current study, to minimize the direct stimulatory effects of leptin on breast cancer cells [33], we washed the leptin-treated M2 macrophages extensively before loading them in the transwell co-culture system to analyze breast cancer cell migration and invasion. Nevertheless, residual leptin, which may remain on the M2 macrophages even after extensive wash, could diffuse into the chamber of breast cancer cells and directly induce cancer cell migration and invasion. However, our experiments *in vivo* showed that depleting mouse macrophages and neutralizing mouse IL-8 significantly reduced leptin-induced xenograft growth and metastasis, which clearly support that macrophage-derived IL-8 can contribute to leptin-induced breast cancer progression.

IL-8, a pro-inflammatory factor, has been shown to promote cancer progression not only by directly stimulating cancer cell proliferation, survival, and migration but also by increasing angiogenesis [31, 32]. Our current study support that at least one of the sources of IL-8 appears to be the tumor microenvironment. Notably, our IHC staining of human specimens suggest that the proportions of cells with positive staining for the leptin receptor ObR, the macrophage marker CD68, and IL-8 increase as breast cancer progresses from carcinoma *in situ* to invasive carcinoma. Thus, elevation of the leptin-ObR-IL-8 axis in breast cancer may actually reflect cancer progression. The leptin-ObR-IL-8 axis may be also one of the mechanisms underlying obesity-induced breast cancer progression.

In summary, our study suggest that leptin may promote breast cancer progression by stimulating IL-8 production in M2 macrophage. The leptin-ObR-IL-8 axis certainly adds another layer of complexity in the tumor microenvironment-breast cancer interaction, which drives cancer progression.

## MATERIALS AND METHODS

### Ethics statement

The protocol for laboratory animal handling and experiments was in strict accordance with Chongqing Management Approach of Laboratory Animal (Chongqing government order NO.195). The protocol has been approved by the Institutional Review Board of Chongqing Medical University (Reference Number: CQMU 2010–26). Surgery on animals was performed under sodium pentobarbital anesthesia, and all efforts were made to minimize animal suffering.

### Animals

Female nude BALB/c mice (6–8-week old) were purchased from the Center of Laboratory Animals of Chongqing Medical University (Chongqing, China). All the mice were monitored daily by animal care staff and laboratory personnel. The mice had free access to food and water and were properly treated if distress, infection, or inflammation occurred. When aggregate tumor burden exceeded 1 cm in diameter or body condition score was < 2, the mice were euthanized by CO_2_ narcosis. We used a regulated flow valve (in addition to cylinder regulator) to control the CO_2_ flow rate to 20% of chamber volume per minute. These methods were chosen because they caused minimal stress to the animals. The euthanasia procedure was consistent with the recommendations of the Panel on Euthanasia of the American Veterinary Medical Association.

### Culture cells

THP1 human monocyte line, human breast cancer cells MCF7 and MDA-MB-231, and mouse macrophage cells RAW264.7 were obtained from the American Type Culture Collection (ATCC) (Rockville, MD, USA). THP1 cells, MDA-MB-231 cells, and RAW264.7 cells were cultured in RPMI 1640; MCF7 cells were kept in Dulbecco's Modified Eagle Media (DMEM). The culture media were supplemented with 10% fetal bovine serum, penicillin (100 U/mL), and streptomycin (100 μg/mL).

### M2 macrophage induction

To induce macrophage differentiation (THP1 macrophages), THP1 cells (1 × 10^6^ cells per well) were cultured in 6-well plates and treated with PMA (100 nM) for 72 hours. To induce M2 phenotype, THP1 cells were treated with PMA (100 nM) for 36 hours and then with PMA (100 nM) and IL-4 (20 ng/mL) for additional 36 hours. The morphology of these cells was observed; phase contrast images of the cells were collected under an inverted microscope.

### Flow cytometry

THP1 cells were washed and re-suspended in PBS after PMA or PMA + IL-4 induction. To determine the expression of CD206, the cells were then incubated with PE-labeled anti-CD206 antibody (BioLegend, San Diego, CA, USA). To determine the intracellular expression of IL-10, IL-12, and TGF-β, the cells were fixed with 4% paraformaldehyde, permeabilized with 0.5% Triton X-100 (BioLegend), and stained with PE-labeled anti-IL-10, anti-IL-12, anti-TGF-β antibodies (BioLegend). After final wash, the labeled cells were analyzed by flow cytometry on a FACScan flow cytometer (BD Biosciences).

### Immunofluorescence staining and confocal microscopy

THP1 cells, THP1 macrophages, and M2 macrophages (3 × 10^5^ cells/well) were incubated with primary antibodies against ObR (Santa Cruz), washed, and then incubated with FITC-conjugated goat anti-rabbit IgG (Beyotime, China). The nuclei were stained with DAPI. Fluorescence images were collected using an Eclipse 80i Microscope (Nikon, Japan).

### Quantitative real-time PCR

Total RNA was extracted using the TRIzol reagent (Invitrogen, USA) and reverse transcribed into first-strand complementary DNA (Invitrogen) according to the manufacturer's instruction. Quantitative real-time reverse transcription PCR (qRT-PCR) was performed according to the standard protocol of the SYBR Premix Ex Taq^TM^ kit (TaKaRa, Japan). All the reactions were performed in a volume of 25 μL in triplicate. Primers for ObRb, ObRt, CCL2, CCL18, CCL17, IL-8, TNF-α, IL-6, IL-10, MMP9, TGF-β1, and β-actin were obtained from Invitrogen. The primer sequences are displayed in Table 1. The PCR reaction condition was: initial denaturation at 95°C for 30 seconds and 40 cycles of denature at 95°C for 5 seconds, annealing at 60°C for 20 seconds, and extension at 72°C for 30 seconds. Relative gene expression of the target gene to β-actin was calculated by normalizing the target gene level to β-actin gene level. The second derivative maximum method was used to determine the Crossing point (Cp) for each sample (in triplicate). The mean value was used to calculate the ΔCp (Cp of the target gene - Cp of β-actin gene). Expression levels of genes were obtained according to the equation: 2^−ΔΔCp^.

**Table 1:** Primer sequence for qRT-PCR analysis of cytokines from M2 macrophages

Gene	Forward primer 5′-3′	Reverse primer 5′-3′	Amplicon (bp)
ObRb	CAGAAGCCAGAAACGTTTCAG	AGCCCTTGTTCTTCACCAGT	344
ObRt	CATTTTATCCCCATTGAGAAGTA	CTGAAAATTAAGTCCTTGTGCCCAG	273
CCL2	AAGATCTCAGTGCAGAGGCTCG	CACAGATCTCCTTGGCCACAA	103
CCL18	CTCTGCTGCCTCGTCTATACCT	CTCTGCTGCCTCGTCTATACCT	108
CCL17	AGGGACCTGCACACAGAGAC	CTCGAGCTGCGTGGATGTGC	133
IL8	ACTCCAAACCTTTCCACC	CTTCTCCACAACCCTCTG	155
TNFα	CTGGGCAGGTCTACTTTGGG	CTGGAGGCCCCAGTTTGAAT	272
IL6	TGCAATAACCACCCCTGACC	GTGCCCATGCTACATTTGCC	163
IL10	AGAACCAAGACCCAGACATCA	GCATTCTTCACCTGCTCCAC	139
MMP9	CGGAGCACGGAGACGGGTAT	TGCAGGCGGAGTAGGATTGG	390
TGF-β1	CATCAACGGGTTCACTACC	CTCCGTGGAGCTGAAGCA	166
β-actin	CACGATGGAGGGGCCGGACTCATC	TAAAGACCTCTATGCCAACACAGT	290

### Western blot analysis

M2 macrophages were lysed in RIPA lysis buffer (Beyotime) and the protein concentration in the cell lysate was determined by BCA method. A total of 80 μg of M2 macrophage cell lysate was separated by SDS-PAGE. The proteins were then transferred to a PVDF membrane, and the membrane was incubated with the primary antibodies against human ObR (Santa Cruz), IL-8 (Abcam), pSTAT-Tyr^705^, pAKT-Ser^473^, pERK-Thr^202^/Tyr^204^, STAT3, AKT, ERK (Cell Signaling Technology, USA), and β-actin (Santa Cruz). HRP anti-mouse or rabbit IgG (Beyotime, China) was used as the secondary antibodies. The antigen-antibody reaction was visualized by enhanced chemiluminescence (ECL) assay (VIAGENE, USA).

### Migration assay (scratch assay)

Breast cancer cell migration was evaluated by the transwell co-culture system (porous polycarbonate membrane filters with 0.4 μm pore, Millipore, USA). MCF7 and MDA-MB-231 cells were cultured to confluence in the lower chamber of the 6-well plates, and a sterile pipette tip was used to scratch across the cell layer. M2 macrophages were induced with leptin (100 ng/mL, Peprotech) for 12 hours and then the M2 macrophages were washed with DMEM to remove leptin. After wash, the M2 macrophages (1 **×** 10^6^ cells/mL per chamber) were added into the upper chamber. The M2 macrophages and breast cancer cells were co-cultured for 48 hours. Phase contrast images of the breast cancer cells were collected under a microscope.

### Invasion assay

Breast cancer cell invasion was evaluated by a 24-well transwell co-culture system (Millipore, USA) with 8 μm-pore polycarbonate filter membrane coated with 3.5 mg/mL matrigel (Sigma, USA). Breast cancer cells (2 × 10^4^ cells/mL per well) were cultured in the upper chamber. M2 macrophages were treated with leptin (100 ng/mL) for 12 hours, collected, and washed. The M2 macrophages (2 × 10^6^ cells/mL per well) were then added in the lower chamber. Breast cancer cells and M2 macrophages were co-cultured for 24 hours. Breast cancer cells inside the chamber were removed by cotton swabs and the cells passing through the membrane of the upper chamber were stained with 0.1% crystal violet for 10 minutes and counted under a microscope. A total of 5 to 10 observation views were randomly selected on each membrane and the average number of cells was calculated.

### Immunohistochemistry of human breast tissue specimens

Tissue sections of human breast tissue specimens were incubated with the antibodies against human ObR, CD68, and IL-8 (Beyotime, China). Cells that showed positive staining for ObR, CD68, and IL-8 were counted. A minimum of 10 observation views at 400× magnification were randomly selected from each tissue section. The average percentage of cells with positive staining was calculated.

### Luciferase reporter assay

Plasmid pGL3-basic was a generous gift from Dr. Qin Zhou (Department of Laboratory Medicine, Key Laboratory of Diagnostic Medicine, Ministry of Education Chongqing Medical University, Chongqing, China). A total of 1 **×** 10^5^ M2 macrophages were seeded in 24-well plates and co-transfected with a total of 800 ng of plasmid pGL3-basic-IL8 and the control plasmid pGL3-basic using Lipofectamine 2000 (Invitrogen). After 48 hours of culture, the cells were lysed and the lysates were collected. Renilla and firefly luciferase activities in the cell lysate were measured with a Dual-Luciferase Reporter System (E1910, Promega, WI, USA).

### Isolation of mouse peritoneal macrophages

Mice were injected intraperitoneally with 1 mL 3% thioglycollate broth (Sigma-Aldrich) to induce an inflammatory response. Three days later, the mice were injected intraperitoneally with 5 mL cold PBS to harvest macrophages. Based on our preliminary experiments, this protocol can yield a larger number of inflammatory macrophages. Approximately 1 × 10^7^ macrophages can be collected from one mouse. Macrophages were isolated from peritoneal lavage by plastic adherence.

### Tumor xenograft

MDA-MB-231 cells (1 × 10^7^ cells for each mouse) were inoculated into the mammary fat pad of female nude mice (6-week old, *n* = 5 per group). When the diameter of the xenograft tumor reached 0.5 cm, mice were randomized into PBS and leptin groups. Mice in the PBS group received intratumor injection of PBS; mice in the leptin group received leptin injection in the tumor (0.1 μg/g) once every 5 day for 4 consecutive weeks. To induce macrophage depletion, mice were injected intraperitoneally with Clophosome^®^-clodronate liposome (neutral) or control neutral liposome at an initial dose of 0.2 mL per mouse and then 0.1 mL per mouse once a week for 8 weeks before leptin or PBS injection. Body weight and tumor volume were measured every 5 days. Tumor volume was calculated according to the following equation: V = (L ×W^2^) × 0.5, where “L” and “W” represent the length and width of xenografts, respectively. Mice were sacrificed at day 30 after cancer cell transplantation. Tumor xenografts, lungs, and livers of the mice were dissected; the tissues were analyzed by immunohistochemical staining and H & E staining. The remaining mice were euthanized by CO_2_ asphyxiation on day 60 or when the xenografts reached 1.0 cm in diameter. Immunohistochemical staining of Ki-67 (CapitalBio, China), IL-8, and CD68 of tumor tissue sections was carried out according to the manufacturer's protocol. H&E staining of the lung and liver tissue sections was performed to examine pulmonary and hepatic metastasis.

In the experiment of testing anti-mouse IL-8 antibody, mice in the leptin group were injected intraperitoneally with polyclonal rabbit anti-mouse IL-8 neutralizing antibody (Abbexa, Cambridge, UK) at an initial dose of 0.2 mL (45 μg/mL) per mouse and then 0.1 mL per mouse twice per week for 2 weeks and polyclonal rabbit IgG (Bioss) was used as the isotype control. The antibody injection was carried out when leptin or PBS injection started. Mice received 2 weeks of injection of leptin and the antibodies.

### Statistical analysis

Statistical analyses were performed using SPSS for Windows version 17.0. Student's *t-test* was used to compare 2 groups. All of the data are expressed as mean ± SD. *P-value* < 0. 05(*), 0. 01(**), or 0. 001(***) was considered as statistically significant.

## Abbreviations

AKT: Protein Kinase B
AMPK: Adenosine Monophosphate Activated Protein Kinase
CCL: CC chemokine ligand
ERK: Extracellular signal-regulated kinase
HE: Hematoxylin and eosin
IFN: Interferon
IHC: Immunohistochemistry
IL: Interleukin
JAK: Junas Kinase
JNK: Jun kinase
LPS: Lipopolysaccharide
MAPK: Mitogen-activated protein kinase
MMP: Matrix metalloproteinase
ObR: Ob receptor
PI-3K: Phosphatidylinositol-3 kinase
PKC: Protein Kinase C
PMA: Phorbol esters
STAT: Signal transduction and activators of transcription
M2 macrophage: Tumor associated macrophages
TGF: Transforming growth factor
TNF: Tumor necrosis factor
VEGF: Vascular endothelial growth factor
